# Endovenous Laser Ablation of Insufficient Superficial Veins in Patients After Deep Vein Thrombosis Recanalisation

**DOI:** 10.1155/cdr/8458282

**Published:** 2025-10-14

**Authors:** Srdjan Babic, Jovan Petrovic, Masa Petrovic, Slobodan Pesic, Slobodan Tanaskovic, Ana Cvetkovic, Predrag Gajin, Nenad Ilijevski

**Affiliations:** ^1^Department of Vascular Surgery, Vascular Surgery Clinic, Institute for Cardiovascular Diseases “Dedinje”, Belgrade, Serbia; ^2^Faculty of Medicine, University of Belgrade, Belgrade, Serbia; ^3^Department of Cardiology and Internal Medicine, Vascular Surgery Clinic, Institute for Cardiovascular Diseases “Dedinje”, Belgrade, Serbia; ^4^Institute for Cardiovascular Diseases “Dedinje”, Belgrade, Serbia; ^5^Department of Anaesthesia and Intensive Care Unit, Surgical Oncology Clinic, Institute for Oncology and Radiology of Serbia, Belgrade, Serbia

**Keywords:** deep vein thrombosis, endovenous ablation, great saphenous vein, thrombophilia, venous insufficiency

## Abstract

**Introduction:**

This study is aimed at investigating the safety of endovenous laser ablation treatment in the superficial venous system and perforator venous system in patients with previous deep vein thrombosis.

**Methods:**

From February 2017 to January 2023, 28 patients (20 women) with an average age of 41.5 ± 8.6 years, insufficient great saphenous vein and previous deep vein thrombosis were treated. All patients were diagnosed based on previous ultrasound examinations. In this retrospective study, patients were indeed included if they met specific criteria. Preoperative evaluations and thrombophilia assessments were conducted.

**Results:**

Most patients had previous thrombosis in the popliteal vein (36.4%), while 31.8% had thrombosis in both the femoral and calf veins. Technical success was achieved in all patients, with concomitant procedures (closure of incompetent perforators and phlebectomies), and during follow-up (20 ± 16 months, range: 2–168 months), there were no signs of recanalisation of previously treated superficial veins. Patients were followed up at 1-month, 3-month, 6-month and 12-month intervals postprocedure. Recurrent deep vein thrombosis occurred in two (7%) patients after 26 and 31 months after treatment. Early complications included one (3.5%) case of endovenous heat-induced thrombosis Type II.

**Conclusions:**

Endovenous laser ablation is safe and effective in patients with resolved deep vein thrombosis when combined with appropriate anticoagulant therapy.

## 1. Introduction

Great saphenous vein (GSV) reflux plays a crucial role in developing chronic venous insufficiency, leading to a range of signs and symptoms [1]. Various treatment options are available to address GSV incompetence, including traditional surgery, endovenous laser ablation (EVLA), radiofrequency (RF) ablation and ultrasound-guided foam sclerotherapy. Recent advancements include cyanoacrylate glue, mechanochemical ablation and endovenous steam ablation [2–4].

While EVLA has proven to be effective, there have been reports of deep venous thrombosis (DVT) occurring in approximately 0.3%–1% of cases involving GSV ablation [3, 5–7]. Past studies have highlighted a history of venous thromboembolic events as a potential risk factor for DVT following endovenous GSV ablation [4, 8–11]. This brings forth a complex challenge in phlebology when faced with varicose veins and coexisting DVT, emphasising the need for a nuanced approach to treatment. Despite its potential, the application of EVLA in patients with a history of DVT remains an area of inquiry [10, 12, 13]. Literature remains scarce on EVLA in patients with a history of DVT, and thus, it is important to further shed light on patient selection criteria, procedural success rates, associated complications and follow-up outcomes in this patient group to update guidelines and improve clinical management of these patients.

The study is aimed at aiding in the effective management of these patients, while closely following the most recent treatment guidelines [14].

## 2. Materials and Methods

### 2.1. Patients

During the period from February 2017 to January 2023, a comprehensive cohort of 28 patients (20 women) with an average age of 41.5 ± 8.6 years was included in this retrospective study. The selection criteria encompassed patients with insufficient GSV and perforating veins, varicose veins and a history of previous DVT. The inclusion criteria were as follows: (1) patients with a well-documented history of lower extremity venous thromboembolic events; (2) patients who were treated for DVT at our institution, with available medical records documenting the event; (3) patients who had a history of DVT and were treated with standard anticoagulation; (4) patients who experienced both DVT and pulmonary embolism (PE); (5) patients who had a history of venous clot and duplex evidence of postthrombotic changes in the examined deep veins and (6) patients with symptomatic superficial vein incompetence after deep vein thrombosis resolving. Patients who met all six criteria were included. Preoperative and postoperative duplex scans were performed by an experienced vascular surgeon using high-resolution colour duplex scanners. Veins were examined in the transverse and longitudinal views. Venous segments were considered incompetent if reversed flow lasted more than 0.5 s in the standing position after manual compression and release. Interrogation of the superficial and deep venous systems included the GSV, small saphenous vein (SSV), perforating veins, common femoral vein (CFV), femoral vein, popliteal vein and calf veins.

### 2.2. Laboratory Examination

Thrombophilia assessment was conducted for all enrolled patients, thereby revealing a constellation of diverse predisposing factors. Detailed laboratory findings are shown in Table 1.

### 2.3. Drug Administration

During DVT treatment, patients were managed with oral anticoagulant therapy for 6–12 months. After confirming the absence of DVT through ultrasound, rivaroxaban was administered at a dose of 2.5 mg twice daily or 10 mg once daily 3 months before the intervention [15, 16]. The median time between DVT onset and EVLA was 10.1 ± 1.2 months. In patients who were allergic to rivaroxaban or had other side effects, an alternative was given. Five days before the intervention, rivaroxaban was switched to low-molecular-weight heparins (LMWHs). The dosage of LMWHs was determined based on body weight (approx. 1 mg/kg once daily for prophylactic anticoagulation using enoxaparin), renal function (adjustment for CrCl < 30 mL/min) and balance of risk of thrombosis versus bleeding (for patients with confirmed factor V Leiden). After the procedure, LMWHs were continued for 7 days, followed by a resumption of rivaroxaban. Patients with specific thrombophilia risk factors (factor V Leiden mutation, prothrombin gene mutation, protein C deficiency, protein S deficiency and Antithrombin III deficiency) were managed differently to address their individual needs. All patients provided written informed consent, and the Ethical Committee of the Institute for Cardiovascular Disease “Dedinje,” University of Belgrade, Serbia, approved the study (Approval No. 2628).

### 2.4. Follow-Up

All patients underwent EVLA, and a postoperative follow-up was conducted at specific intervals. Patients were seen by the attending surgeon, and clinical evaluation and duplex scanning were performed on the 1st, 7th and 30th days after surgery. Additionally, patients were asked to return for follow-up visits at 6- and 12-month post-op during the first year and annually thereafter. The average follow-up period was 20 ± 16 months (ranging from 2 to 168 months). The follow-up protocol included duplex evaluation of the treated segment, CFV, femoral vein, popliteal vein and calf veins. The study endpoint was the obliteration of GSV and perforating veins and the absence of DVT in any lower extremity segment. Given their elevated risk, patients with thrombophilia received closer follow-up post-EVLA. Ultrasound surveillance of the treated veins was conducted more frequently to detect early signs of thrombosis. Compression stockings and lifestyle modifications were also emphasised to support venous circulation.

### 2.5. Technique

#### 2.5.1. Anaesthesia and Sedation

Tumescent anaesthesia, consisting of 500 mL of normal saline mixed with a 20 mL solution of 2% lidocaine, 1:100,000 epinephrine and 4 mL of 7.5% sodium bicarbonate solution, was infiltrated around the target vein. This was done to achieve vein wall apposition and reduce heat dissipation to the perivenous tissues. Before the procedure, occasional oral sedation was administered (15 mg of midazolam taken orally 15 min before the intervention).

#### 2.5.2. Laser Procedure

The procedures were performed by a single experienced vascular surgeon (performed over 10,000 procedures) in collaboration with a physician, following a standardised procedure protocol. The GSV was punctured under ultrasound guidance below knee level, and an introducer sheath was advanced over a J wire. The sheath was replaced with a catheter, which was then positioned in the GSV, and a fibre (laser energy 1064 nM) was placed 1 cm distal to the superficial epigastric vein. The perforating vein was punctured under ultrasound, and the fibre was placed at the level of the superficial fascia. EVLA of the saphenous and perforating veins was performed using laser ablation (Fotona's XP-2 family, Nd:YAG laser, VSP, Ljubljana, Slovenia). Varicose veins were removed through skin microincisions using Mueller or Ramelet-style hooks.

#### 2.5.3. Postoperative Care

Patients were encouraged to remain active and wear 20–30 mm graduated strength compressive stockings 24 h a day for the first 7 days and then for 2 weeks during the day. Postoperatively, anticoagulation was restarted promptly to prevent recurrence of DVT. The first prophylactic dose of LMWH (1 mg/kg once daily) was administered 1 h after surgery and continued for 3 days postoperatively. Patients with thrombophilia were typically maintained on therapeutic anticoagulation (e.g., rivaroxaban, apixaban or continued LMWH) for an extended period compared to other patients, sometimes for several months after the procedure. In cases of specific thrombophilias, such as antiphospholipid syndrome, where the risk of thrombosis is particularly high, warfarin was resumed. For others, such as those with heterozygous factor V Leiden, rivaroxaban was preferred due to its convenience and lower bleeding risk profile.

## 3. Results

Patients' characteristics are presented in Table 2. The study population had a mean age of 41.5 ± 8.6 years, with a female predominance (71.7%). Diabetes mellitus was present in 7.1% of the patients, hypertension in 3.6% and obesity in 7.1%. A significant portion of the patients were smokers (39.3%). Unilateral procedures were performed on all the patients. Regarding DVT, the popliteal vein was the most commonly affected proximal vein, found in 36.4% of cases, while 31.8% had femoral vein involvement, and 31.8% had calf vein affected. According to the CEAP classification, 71.7% of patients were classified as C2–C4, while 28.3% were classified as C5–C6. Two (7.1%) patients had active venous ulcers. The mean proximal GSV diameter, measured at 3 cm below the SFJ and midthigh in the standing position, was 13.1 ± 2.8 mm. The EVLA technique yielded a commendable success rate of 100% within the cohort. Notably, 89.3% of the patients underwent concurrent procedures, encompassing phlebectomy and transluminal occlusion of perforators (TRLOPs)—a minimally invasive procedure aimed at blocking or closing perforating veins, which connect the superficial venous system to the deep venous system in the legs using laser ablation (Table 2). During follow-up (20 ± 16 months, range: 2–168 months), there were no signs of recanalisation of previously treated superficial vein. No patients exhibited symptoms of postthrombotic syndrome, and all previous DVTs were recanalised. During the follow-up period, recurrent DVT occurred in two patients, representing 7% of the cohort. These events were observed at 26- and 31-month postprocedure in patients with known thrombophilia (one with factor V Leiden and one with a prothrombin gene mutation). The spectrum of complications was relatively low, comprising a sole case of endovenous heat-induced thrombosis (EHIT) Type II and incidental occurrences of minor skin haematomas, which did not warrant any intervention.

## 4. Discussion

In the absence of a clear consensus in guidelines, our study is aimed at presenting a cohort of patients at high risk for thromboembolism, evaluating the safety and efficacy of EVLA in these patients, while emphasising the importance of carefully tailored management strategies to ensure the best possible outcomes. The coexistence of deep and superficial venous pathology is regarded as a relative contraindication for performing superficial venous interventions. This perception is especially pronounced in patients with a history of DVT, where the assumption exists that addressing reflux in superficial veins may impair venous drainage and exacerbate the symptoms of chronic venous disease [11]. According to the guidelines published by the European Society for Vascular Surgery, in patients with chronic venous disease caused by combined superficial and deep venous incompetence, treatment of incompetent superficial veins should be considered (Class IIa, Level C) [11]. Adam et al. showed that in more than 50% of cases, superficial vein treatment can result in the correction of segmental deep venous reflux [17]. The venous clinical severity score has shown significant improvement following the EVLA of incompetent saphenous veins in patients presenting with both superficial and deep vein reflux [13, 18]. Finally, in patients who have superficial reflux alongside deep vein obstruction, the GSV seldom serves as a collateral vessel, and it does not contribute to lower limb drainage if there is reflux present [11].

The recurrence rate of DVT observed in our study, at 7%, occurring at 26- and 31-month post-EVLA, is consistent with findings from other studies in the field. Notably, Di Gangi et al. reported a 7.2% incidence of DVT following EVLA, closely mirroring our results [19]. However, a key distinction is that only 0.3% of the patients in their cohort had a prior history of DVT, whereas in our study, the recurrence was exclusively observed in patients with known thrombophilia. This difference highlights the higher risk profile present in our patient population, suggesting that pre-existing conditions like thrombophilia significantly influence DVT recurrence rates postprocedure.

Furthermore, Di Gangi et al. found that the continuation of preoperative anticoagulation therapy did not significantly impact the rates of DVT following EVLA, with similar incidence rates observed between those who received posttreatment prophylaxis and those who did not (8.2% vs. 9.5%) [19]. This observation aligns with our findings, where anticoagulation status did not appear to affect the recurrence rates of DVT in our cohort, highlighting the potential limitations of anticoagulation in preventing DVT in this context.

Contrasting these results, data from the National Vascular Quality Initiative Varicose Vein Registry assessed by Chervonski et al. provided different insights. Their study, which included 33,892 endovenous thermal ablations performed on 23,572 individual patients, found that a history of DVT significantly increased the risk of new DVT (1.4% vs. 0.8%; *p* = 0.030), proximal thrombus extension (2.3% vs. 1.6%; *p* = 0.045) and bleeding complications (0.2% vs. 0.04%; *p* = 0.030) following EVLA [20]. These findings stand in contrast to our study, where no significant differences in these complications were observed. This discrepancy may suggest that the tailored management approach implemented in our cohort could have contributed to mitigating these risks, emphasising the importance of individualised patient care in high-risk groups.

Demographic differences between our study and that of Chervonski et al. might also play a role in the differing outcomes observed. Our cohort predominantly consisted of female patients with a mean age of 41.5 years, while the Chervonski et al. study had a more balanced gender distribution with a slight predominance of male patients and a higher mean age [20]. These variations in demographics are clinically relevant, as older age and male gender are well-established risk factors for thrombotic events, which could partially explain the higher incidence of complications reported in their study.

Additionally, it is noteworthy that the CEAP classification in the Chervonski et al. study was similar to our population's distribution, with both cohorts predominantly including patients classified as C2–C4. This similarity reinforces the relevance of our findings in the context of chronic venous disease severity, indicating that despite differences in demographics and treatment approaches, the baseline venous pathology severity was comparable between the two studies [20].

Recurrent DVT remains a major clinical challenge. Recent studies have shown that it is a multifactorial condition, with long-term factors playing a key role in recurrence. Effective anticoagulation is essential to reduce the risk of recurrent VTE events, not only in the immediate postprocedural period but also later on, indicating that persistent risk factors, underlying thrombophilia, or insufficient long-term anticoagulation may significantly contribute to recurrence. In this context, Chervonski et al. reported a 1.4% risk of early postprocedural DVT within 3 months for patients with a history of DVT compared to 0.8% for those without such a history; our study did not observe a similar increase, potentially due to our focus on customised patient management strategies [20].

Our study's high procedural success rate aligns with the findings of Proebstle et al. and Lurie et al., who reported successful endovenous RF obliteration in selected patient populations [21, 22]. However, the notable scarcity of literature addressing endovenous ablation in patients with previous DVT remains evident.

Genetic mutations are known to play a significant role in thrombophilia. One of the most well-known genetic mutations associated with thrombophilia is factor V Leiden. This mutation involves a change in clotting Factor V, resulting in an elimination of the cleavage site in Factor V and Factor Va, making it resistant to inactivation by protein C [23]. Antithrombin deficiency, protein C deficiency and protein S deficiency are inherited thrombophilias caused by mutations affecting these natural anticoagulant proteins, making individuals more susceptible to clot formation [24]. It is important to note that not everyone with these genetic mutations will develop blood clots, and the occurrence of thrombosis often involves a combination of genetic and environmental factors. The identification of these genetic mutations is crucial for individuals with a family history of thrombosis or unexplained clotting events. It allows for risk assessment and tailored preventive measures. Genetic testing can also guide clinical decisions regarding the duration and intensity of anticoagulation therapy [24, 25].

The presence of thrombophilia in our cohort underscores the complex nature of managing patients with a history of DVT. Thrombophilia can significantly contribute to the development of recurrent DVT and influence treatment outcomes [25]. Using oral anticoagulant therapy, such as rivaroxaban, is a pivotal strategy to mitigate the risk of thrombus formation and recurrence [11]. Thrombophilia tendencies in patients with the heterozygous factor V Leiden mutation, homozygous genotype of C677T MTHFR mutation and heterozygous PAI-1 gene 4G/5G mutation emphasise the need for individualised anticoagulant therapy [25]. The tailored approach, observed in this study with the use of rivaroxaban and bridging with LMWHs, aligns with the evolving trend of personalised medicine in phlebology. Initially, a low dose of rivaroxaban (2.5 mg twice daily) was employed for the treatment of peripheral arterial disease [15, 16]. Additionally, it has been demonstrated that rivaroxaban is equally effective as LMWHs in preventing DVT and EHIT [26]. Therefore, we administered rivaroxaban to all treated patients as a DVT prophylaxis, using a low dosage (10 mg) for 3 months [27]. However, for patients with a confirmed factor V Leiden mutation, the dosage was increased based on the haematologist's recommendation.

The therapeutic approach of administering rivaroxaban, followed by a transition to LMWHs in select cases, was carefully chosen to balance the efficacy of anticoagulation with patient safety. This strategy aligns with evidence from previous studies, such as those by Di Gangi et al., which demonstrated that while posttreatment prophylaxis following EVLA may be associated with an increased risk of bleeding, it does not significantly affect the recurrence rate of DVT [19]. This finding emphasises the need to tailor anticoagulation regimens in a way that prioritises patient safety without compromising therapeutic outcomes.

Further support for this approach is provided by the study conducted by Keo et al., which compared the efficacy of rivaroxaban versus fondaparinux for thromboprophylaxis after EVLA [26]. Their results indicated that rivaroxaban was an effective prophylactic agent, highlighting its role in reducing thrombotic events in this clinical setting. These insights informed our decision to employ rivaroxaban initially, followed by a transition to LMWHs in selected cases where prolonged anticoagulation was necessary.

Additionally, data from the VEINOVA (vein occlusion with various techniques) registry, as reported by Keo et al., further elucidates the impact of thromboprophylaxis on DVT incidence. In this registry, patients who received thromboprophylaxis after EVLA experienced a DVT incidence of only 0.8%, compared to a higher incidence of 2.2% in those who did not receive prophylaxis (*p* = 0.135) [28]. This significant reduction in thrombotic events highlights the potential benefits of a structured prophylactic strategy, emphasising the importance of individualised patient care to mitigate the risk of DVT recurrence.

Collectively, these findings suggest that the use of rivaroxaban as the primary anticoagulant, with subsequent tailoring to LMWHs when appropriate, aligns well with contemporary clinical practices. This approach not only addresses the dual objectives of minimising both thrombotic and haemorrhagic risks but also supports a personalised treatment paradigm.

In managing patients with specific thrombophilia undergoing EVLA after a prior DVT, a tailored approach was essential to minimise the risk of recurrent thromboembolism, given their inherent hypercoagulable state. By adapting anticoagulation therapy to each patient's unique risk profile, clinicians can enhance therapeutic outcomes while minimising adverse effects, embodying the principles of precision medicine in the management of venous thromboembolic disease. For these high-risk patients, the process began with a detailed thrombophilia evaluation and review of their thrombotic history. Those with conditions like factor V Leiden, prothrombin gene mutation or antiphospholipid syndrome were identified as high risk, prompting a modification of the standard approach in cases where they were already on long-term anticoagulation. Bridging therapy with LMWH was employed, and compression therapy was initiated immediately after EVLA to reduce the likelihood of clot formation along the treated vein. Given their prothrombotic tendencies, these patients were closely monitored for longer periods immediately postprocedure to detect any early signs of thrombus propagation.

### 4.1. Limitations

Our study has some limitations. The analysis exclusively draws upon a cohort of a small number of individuals admitted to the hospital, thus tempering its generalisability to the broader spectrum of patients encompassing both DVT and superficial vein insufficiency. The generalisability of the findings may be limited given that all procedures were performed by a single experienced surgeon, potentially reflecting outcomes influenced by the surgeon's specific expertise. These results may not be representative of those achieved by surgeons with varying levels of experience. Additionally, the small sample size may reduce the study's power to detect postprocedural DVT events. Moreover, the quality of life of patients was not assessed during the follow-up period. A larger, multicentre study is needed to confirm these findings.

## 5. Conclusions

This study illuminates the application of EVLA in patients with a history of DVT, emphasising the significance of patient selection, individual approaches, detailed laboratory evaluations and detailed examination during follow-up. Although the sample size is limited, the findings underscore the feasibility of this procedure in carefully selected patients, presenting a potential pathway for enhanced management of this challenging subset.

## Figures and Tables

**Table 1:** Laboratory findings of enrolled patients.

**Variable**	**N** **(%)**
Heterozygous factor V Leiden mutation	3 (10.7)
MTHFR mutation	12 (42.9)
Heterozygous protein C deficiency	4 (14.3)
Heterozygous protein S deficiency	4 (14.3)
Heterozygous PAI-1 gene 4G/5G mutation	16 (57.1)
Antithrombin III deficiency	6 (21.4)
Elevated homocysteine levels	4 (14.3)
Elevated lupus anticoagulants	9 (32.1)
Antiphospholipid syndrome	1 (3.6)

Abbreviations: MTHFR–methylenetetrahydrofolate reductase, PAI-1–Plasminogen activator inhibitor-1.

**Table 2:** Demographic and clinical characteristics of enrolled patients.

**Variable**	**Value**
Mean age (years)	41.5 ± 8.6
Female sex	20 (71.7%)
Diabetes mellitus	2 (7.1%)
Hypertension	1 (3.6%)
Obesity (BMI > 30 kg/m^2^)	2 (7.1%)
Smoking	11 (39.3%)
Most proximal veins affected by DVT	
*Femoral*	9 (31.8%)
*Popliteal*	10 (36.4%)
*Calf*	9 (31.8%)
CEAP classification	
*C2–C4*	20 (71.7%)
*C5*	6 (21.2%)
*C6*	2 (7.1%)
Mean proximal GSV diameters (mm)	13.1 ± 2.8
Concomitant procedure	
*Perforator interruption*	14 (56.0%)
*Phlebotomies*	24 (96.0%)
Provoked DVT	3 (10.7%)
Primary DVT	25 (89.3%)
Oral contraceptive usage	3 (10.7%)
Family history of DVT or PE	1 (3.6%)
Ankle circumference reduction (1 month after surgery, mm)	16.3 ± 6.3

*Note:* All values are presented as *n* (%) or mean ± sd, depending on variable type.

Abbreviations: BMI, body mass index; CEAP, clinical, etiological, anatomical and pathophysiological; DVT, deep vein thrombosis; GSV, great saphenous vein; PE, pulmonary embolism.

## Data Availability

The data presented in this study are available on request from the corresponding author due to ethical reasons.
